# *Dracocephalum tanguticum* Maxim Polysaccharide Ameliorate Autoimmune Hepatitis via Regulating TNF and IL‐17 Signaling Pathways

**DOI:** 10.1002/fsn3.71667

**Published:** 2026-03-24

**Authors:** Min Guo, Saixue Wei, Biaobiao Cheng, Xiaodong Li

**Affiliations:** ^1^ Laboratory of Chinese Medicine Gansu Provincial Hospital of Traditional Chinese Medicine Lanzhou China; ^2^ Zhengzhou Aimili Biotechnology Co., Ltd. Luoyang China; ^3^ College of Pharmacy Gansu University of Chinese Medicine Lanzhou China

**Keywords:** autoimmune hepatitis (AIH), *Dracocephalum tanguticum* Maxim (DrT), IL‐17 signaling pathway, polysaccharide, TNF signaling pathway

## Abstract

*Dracocephalum tanguticum* Maxim (DrT) is a well‐known traditional medicinal and edible plant with hepatoprotective effects. In this study, crude polysaccharides of DrT (DrTPs) were obtained using the water extraction‐ethanol precipitation method. Autoimmune hepatitis (AIH) models of both mice and ALM12 cells were produced by ConA. The serum liver function indexes (AST and ALT) were examined by ELISA, and liver tissue pathological changes were observed by HE staining. The hepatoprotective mechanism of DrTP80 was explored by RNA sequencing and verified by detecting the protein expressions using Western blot. As a result, DrTP80 could significantly reduce AST and ALT levels in the injured liver and ALM12 cells. DrTP80 also obviously improved the hepatopathological changes in liver tissue induced by ConA. Furthermore, RNA sequencing detected significant differences in gene expression, and the functions of differential genes were focused on TNF and IL‐17 signaling pathways. Based on these two signaling pathways, 13 differentially expressed genes (Vcam1, Atf6b, Akt1, Irf1, Map2k3, Lcn2, Hsp90ab1, Anapc5, Traf4, Fosl1, Jun, Cxcl5, Nfκbia) among NC, CRC, and FP groups were screened and verified by Western blot. In conclusion, our results demonstrated that DrTP80 can alleviate immune liver damage induced by ConA, and its hepatoprotective mechanism may be related to regulating TNF and IL‐17 signaling pathways. Our findings indicated that DrTP80 could be exploited as a healthy food supplement for the treatment of immune liver injury.

## Graphical abstract

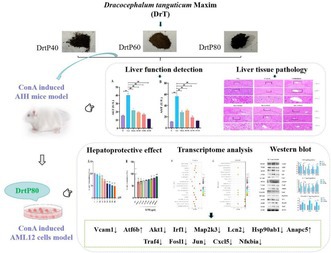

## 1 Introduction

Liver diseases caused by a variety of factors including chemical substances, drugs, viruses, alcohol or immune disregulation remain a health threat worldwide (Devarbhavi et al. 2023). Autoimmune hepatitis (AIH) is characterized by an abnormal immune response against the liver and may develop into severe liver failure or even death with limited therapies (Sebode et al. 2018). The treatment of AIH generally involves the use of steroids alone or in combination with immunosuppressants; however, current therapies often come with significant side effects including disturbed endocrine system and impaired immunity (Richardson et al. 2020). Thus, effective and safe pharmacological treatments are urgently needed. There is accumulating evidence that natural products have great therapeutic potential for liver diseases (Ibrahim et al. 2021; Wen et al. 2025).

*Dracocephalum tanguticum* Maxim (DrT), a well‐known traditional medicinal and edible plant, is mainly distributed in Northeast Asia and the high‐altitude mountain areas in western China (Ministry of Health of the People's Republic of China 1995). DrT has antibacterial, anti‐inflammatory, immunomodulatory and other pharmacological activities, and commonly used for the treatment of hepatitis (Wang et al. 2010; Xu et al. 2011; Ma et al. 2020). Our previous studies have shown that DrT can inhibit the ethanol induced liver injury (Guo et al. 2022).

**FIGURE 1:**
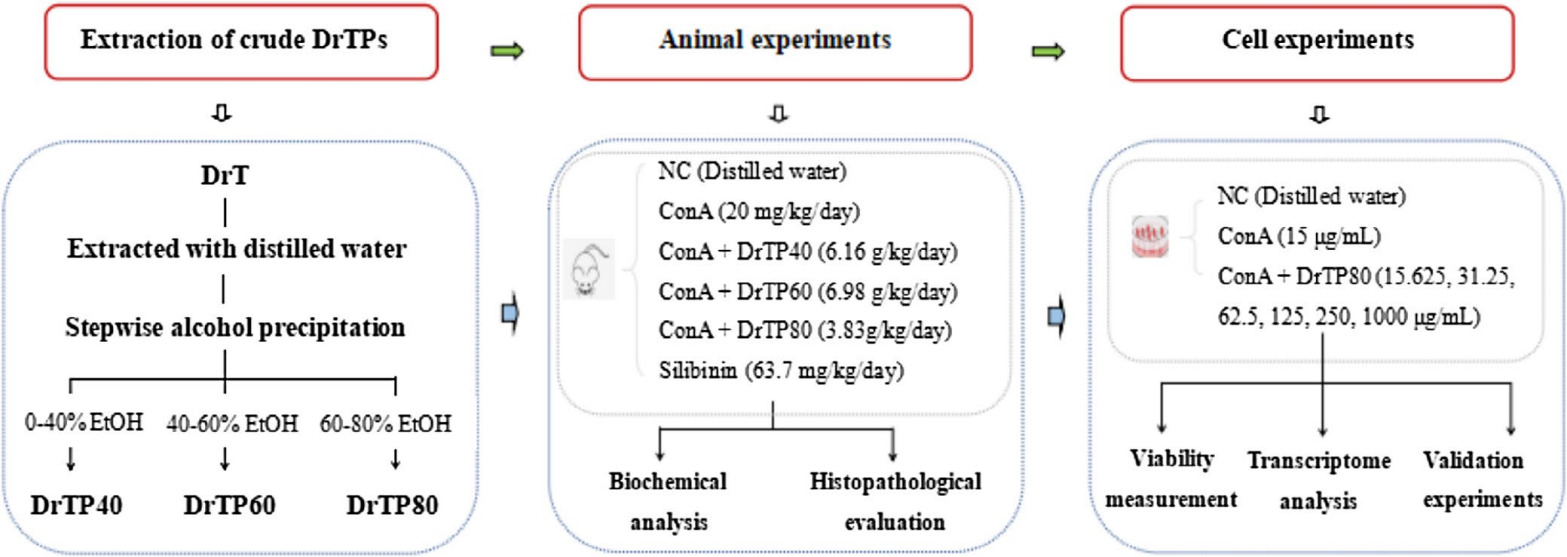
Scheme of the experimental design.

Plant polysaccharides have great potential in ameliorating liver disease (Dedhia et al. 2022; Yang et al. 2020; Bian et al. 2025). Up to now, there has been no scientific report on polysaccharides of DrT (DrTP) in treating liver disease. Therefore, we extracted crude DrTPs (DrTP40, DrTP60, DrTP80) and investigated their hepatoprotective effect against Concanavalin (ConA) induced liver injury in vivo experiments. Furthermore, the study explored the underlying hepatoprotective mechanism of DrTP80 by RNA sequencing in vitro. The experimental design is shown in Figure 1.

## 2 Materials and Methods

### 2.1 Extraction of Crude Polysaccharides From DrT

The DrT was collected from Haidong city in Qianghai Province (101°04′ E; 36°9′ N), which is one of native producing provinces in China, and authenticated by Fangdi Hu, a Professor in Lanzhou University, according to drug standard of ministry of Public Health of the peoples pepublic of China (TibetanMedicine, WS3‐BC‐0023‐95). The specimen is stored in the Laboratory of Chinese Medicine, Gansu Provincial Hospital of Traditional Chinese Medicine (No. 20220811). The DrT powder was removed grease by petroleum ether and the residues were extracted with 20 volumes of water at 90°C for 1.0 h. The supernatant was collected, ethanol was added until its concentration was 40% and centrifuged (3000 r/min, 15 min), the precipitate was collected as DrTP40. Ethanol was added dropwise into the supernatant until its concentration was 60% and centrifuged (3000 r/min, 15 min), the precipitate was collected as DrTP60. Ethanol was added dropwise into the supernatant until its concentration was 80% and centrifuged (3000 r/min, 15 min), the precipitate was collected as DrTP80. DrTP40, DrTP60 and DrTP80 were deproteinated following the sevage method and then lyophilized to obtain the crude DrTPs.

### 2.2 Animal Experiments

#### 2.2.1 Animal and Treatments

Sixty male KM mice (30–32 g) were purchased from Lanzhou University [Production license No. 62000800000320] and housed in an environment of 12‐h light–dark cycles, (22 ± 2)°C and (60 ± 5)% humidity. The design of the animal experiment was approved by the Ethics Committee of Lanzhou Taigu Research Institute (approval No. TGYJY‐2024‐0033).

Mice were randomly divided into normal control group (NC), ConA group, Silibinin group, DrTP40 group, DrTP60 group and DrTP80 group according to body weight, with 10 in each group. Based on the body surface, clinical dosage of DrT (15 g/day) (Ministry of Health of the People's Republic of China 1995) and extraction rate of DrTPs, the Silibinin group, DrTP40 group, DrTP60 group and DrTP80 group were administrated with silibinin (63.7 mg/kg/day), DrTP40 (6.16 g/kg/day), DrTP60 (6.98 g/kg/day) and DrTP80 (3.83 g/kg/day), for 7 days respectively. Except for the NC group, the mice in the other groups were treated with a single dose of ConA (20 mg/kg) to establish the immune liver injury mice model. After an 8‐h fast, all mice were anesthetized, the blood was collected and centrifuged at 3500 rpm for 15 min (3‐18KS; Sigma, USA), the serum was collected and stored at −80°C. The hepatic lobe was fixed in 4% paraformaldehyde for pathological analysis, the rest of the liver tissue was stored at −80°C for gene and protein detection.

#### 2.2.2 Detection Serum Levels of the Liver Function Indexes

The serum levels of AST and ALT were detected by the corresponding kits.

#### 2.2.3 Histopathological Evaluation

The paraformaldehyde fixed liver tissues were paraffin‐embedded, sectioned (5‐μm‐thick), HE stained and observed under a light microscope.

### 2.3 Cell Experiments

#### 2.3.1 ConA Induced Cytotoxicity

AML12 cells were provided by Wuhan Seville Biotechnology Co. Ltd. and kept in DMEM/F12 medium containing 10% (v/v) FBS, 1% (v/v) ITS and 40 ng/mL Dexamethasone sodium phosphate. Cells were seeded into 96‐well plates at a density of 1 × 10^4^ cells/well and placed in a CO_2_ incubator at 37°C for 12 h. Then cells were incubated with ConA (0, 5, 10, 15, 20, 25, 30, 35, 40 μg/mL) for 24 h respectively. After treatments, cells were further incubated with 10% CCK8 for 2 h, and the optical density was measured at 450 and 620 nm.

#### 2.3.2 Effect of DrTP80 on ConA Induced Cells

The cells were divided into nine groups: Normal control group (NC), ConA group, DrTP80 groups (including six groups of DrTP80 with different concentrations). DrTP80 groups were treated with 100 μL of DrTP80 at the concentrations of 0.015, 0.031, 0.062, 0.125, 0.250, and 1.000 mg/mL respectively. All cells were kept in a 5% CO_2_ incubator for 3 h. The cells in ConA and DrTP80 groups were treated with 100 μL of 15 μg/mL ConA for 24 h. After treatments, cells were further incubated with 10% CCK8 for 2 h, and the optical density was measured at 450 and 620 nm.

#### 2.3.3 RNA Sequencing Analysis

Total RNA from cells (NC group, ConA group and DrTP80 1.0 mg/mL group) was extracted. After extraction and fragmentation of mRNA, the first and second strand cDNA were synthesized. The library fragments were constructed and loaded into the Illumina sequencing platform. All data analysis was performed using the NovoMagic cloud platform (magic.novogene.com); this information included Principal components analysis (PCA) and difference analysis. We set *p* value < 0.05 and fold change ≥ 0.5 as the condition for differential gene screening. The functions of these genes and signaling pathways were studied using the KEGG database.

#### 2.3.4 Validation Experiments

The total protein was extracted with cold RIPA buffer. The protein content was determined by the BCA protein assay kit. Separate the protein by electrophoresis and transfer it to the PVDF membrane. The membrane is incubated with primary (β‐actin) and secondary (Vcam1, Atf6b, Akt1, Irf1, Map2k3, Lcn2, Hsp90ab1, Anapc5, Traf4, Fosl1, Jun, Cxcl5, Nfκbia) antibodies, and the location and abundance of the target protein are determined by a colorimetric reaction. The gray densities of the protein bands were normalized and analyzed.

### 2.4 Statistical Analysis

SPSS software was used for data analysis, the results are expressed as mean ± standard deviation. Graphs were generated using Origin Pro. Data were evaluated by One‐way ANOVA with the Tukey HSD post hoc test for comparisons between groups. Results were considered significant for *p* < 0.05.

## 3 Results

### 3.1 Hepatoprotective Effects of DrTPs on ConA Induced Mice

Water‐soluble crude DrTPs were obtained from the original medicinal materials using the water extraction‐ethanol precipitation method. DrTP40, DrTP60, and DrTP80 were extracted from DrT with the yield of 20.38%, 22.68%, and 12.45%.

#### 3.1.1 Results of Serum Liver Function Index Detection

**FIGURE 2:**
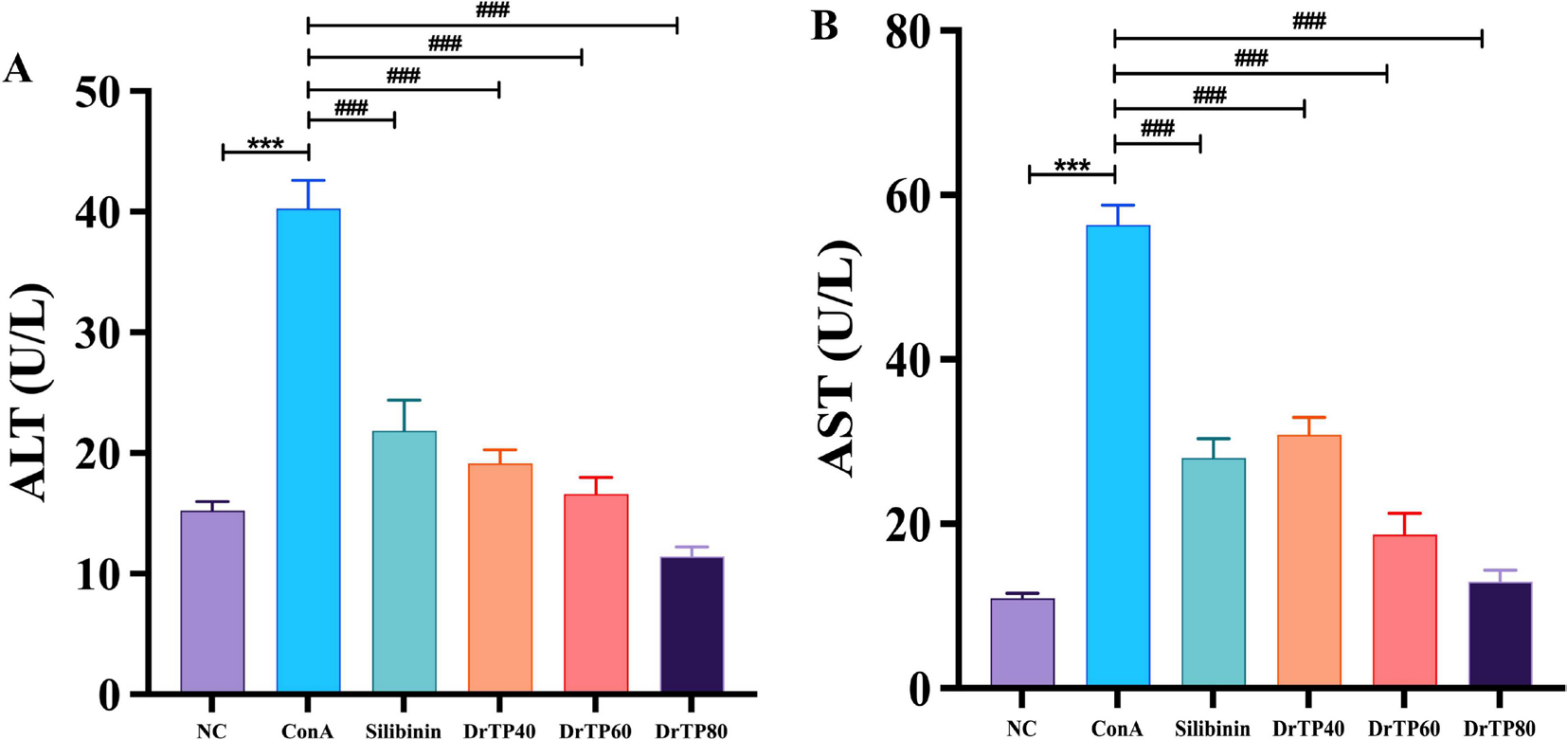
Levels of ALT (A) and AST (B) in serum. All data are shown as mean ± SD (*n* = 10). ***p < 0.001 versus the NC group; ^###^ *p* < 0.001 versus the ConA group. ALT, Alanine aminotransferase; AST, Aspartate aminotransferase; ConA, Concanavalin A.

The results showed that ConA could increase the contents of ALT and AST, which decreased after intervention of silibinin, DrTP40, DrTP60, and DrTP80 (Figure 2).

#### 3.1.2 DrTPs Improves Liver Pathology in AIH Mice

**FIGURE 3:**
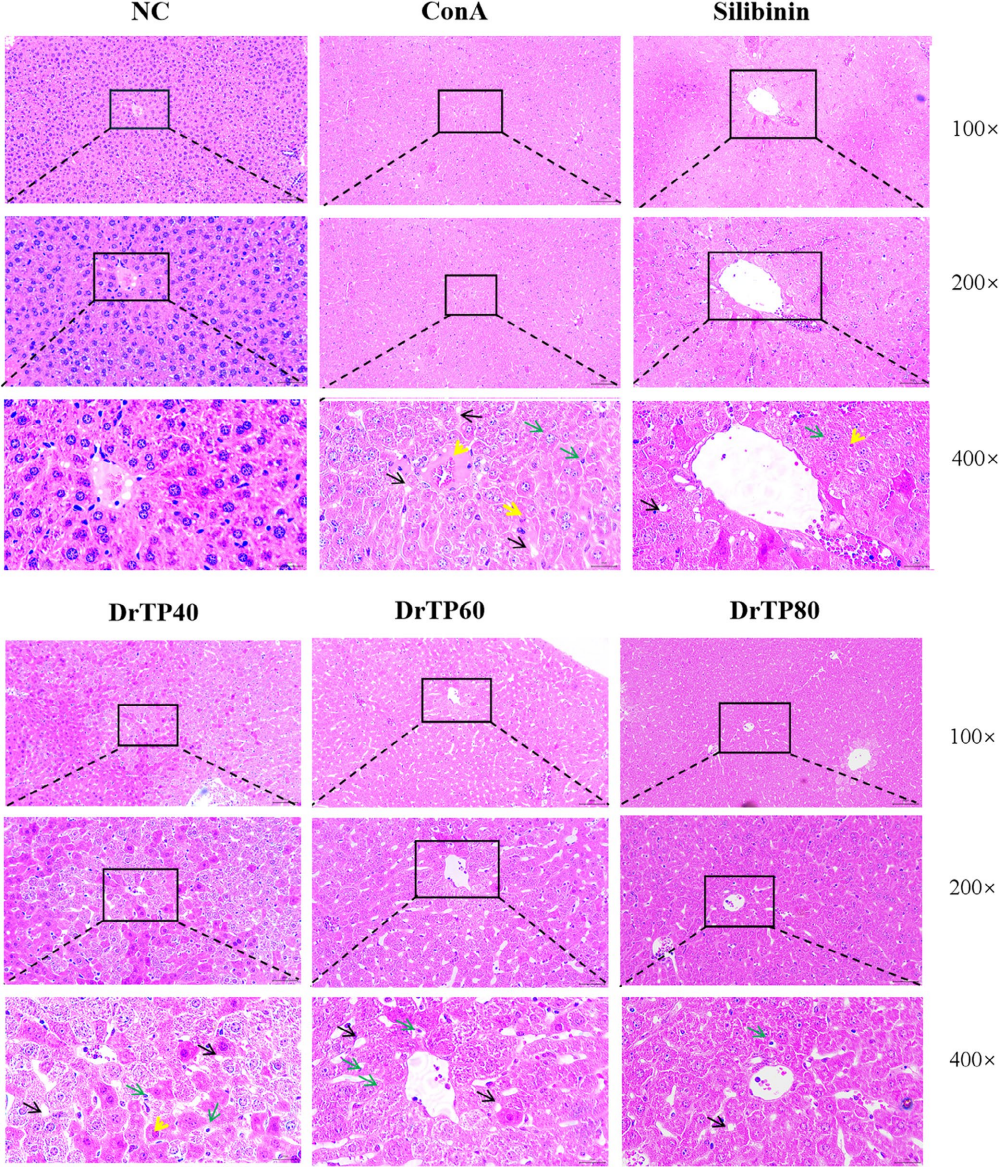
Pathological observation of liver tissue. Hepatocyte ballooning degeneration (black arrow), necrosis (green arrow), and lymphocyte infiltration (yellow arrow).

The results (Figure 3) showed that the hepatic lobules and cells in the NC group were regular. The ConA group exhibited disordered hepatic lobular structure, hepatocyte enlargement, vacuolation, and lymphocyte infiltration. In the DrTP40 group, lobular structure and hepatocyte status were improved. In the DrTP60 group, hepatocytes occasionally had degeneration and spot necrosis. In the silybinin and DrTP80 groups, the pathological structure of the liver was similar to that of the NC group.

### 3.2 Hepatoprotective Effect of DrTP80 on ConA Induced Cells

#### 3.2.1 DrTP80 Inhibits ConA Induced Decrease in Cell Viability

**FIGURE 4:**
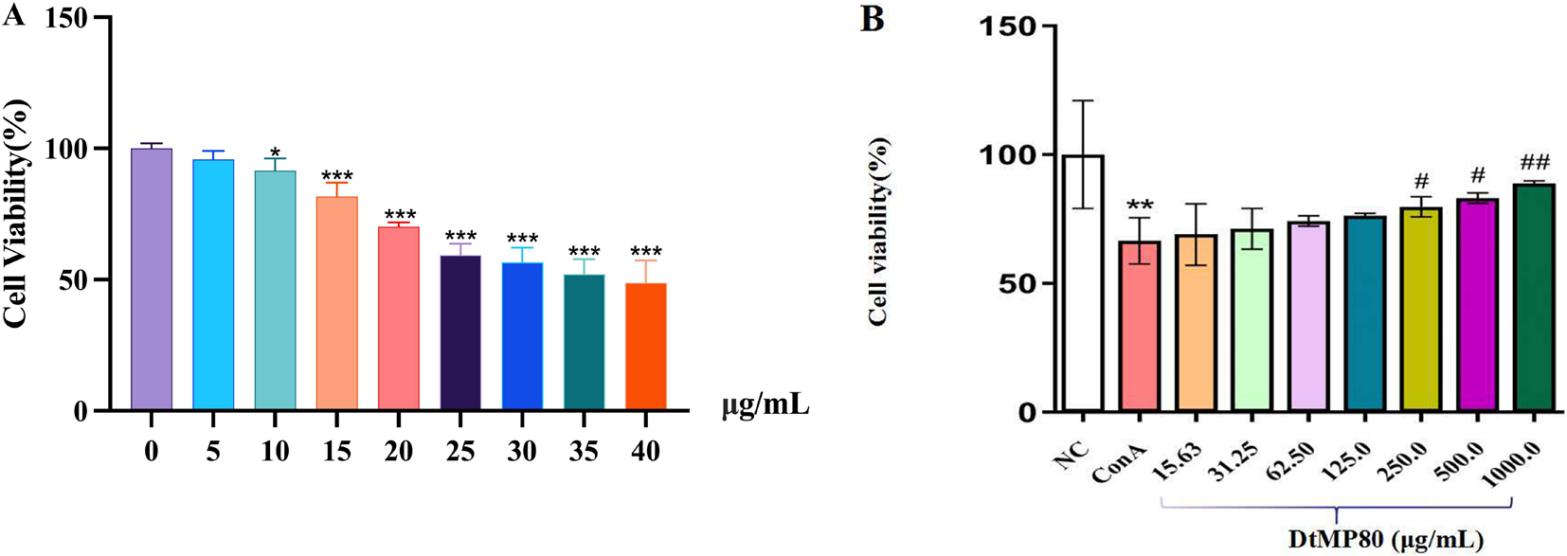
ConA induced cytotoxicity (A) and the effect of DrTP80 on ConA induced cells (B). All data are shown as mean ± SD (*n* = 6). *p < 0.05, **p < 0.01 and ***p < 0.001 versus the NC group; ^#^ *p* < 0.05 and ^##^ *p* < 0.01 versus the ConA group. ConA, Concanavalin A; DrTP80, polysaccharide 80 of *Dracocephalum tanguticum* Maxim.

An immune liver injury cell model was generated by ConA induction. As shown in Figure 4A, in the ConA group, the cell viability had no significant change at the concentrations of 5 (95.66 ± 3.8) and 10 μg/mL (91.43 ± 4.72). While at the concentrations of 15 (81.63 ± 5.31), 20 (70.17 ± 1.6), 25 (59.58 ± 4.32), 30 (56.49 ± 5.69), 35 (51.95 ± 5.76), and 40 μg/mL (48.93 ± 8.47), cell viability was significantly decreased. The results (Figure 4B) showed that at concentrations of 15.63, 31.25, 62.5, and 125.0 μg/mL, the cell viability remained almost unchanged; at concentrations of 0.25, 0.5, and 1 mg/mL, DrTP80 could increase the cell viability.

#### 3.2.2 Transcriptome Analysis Results

**FIGURE 5:**
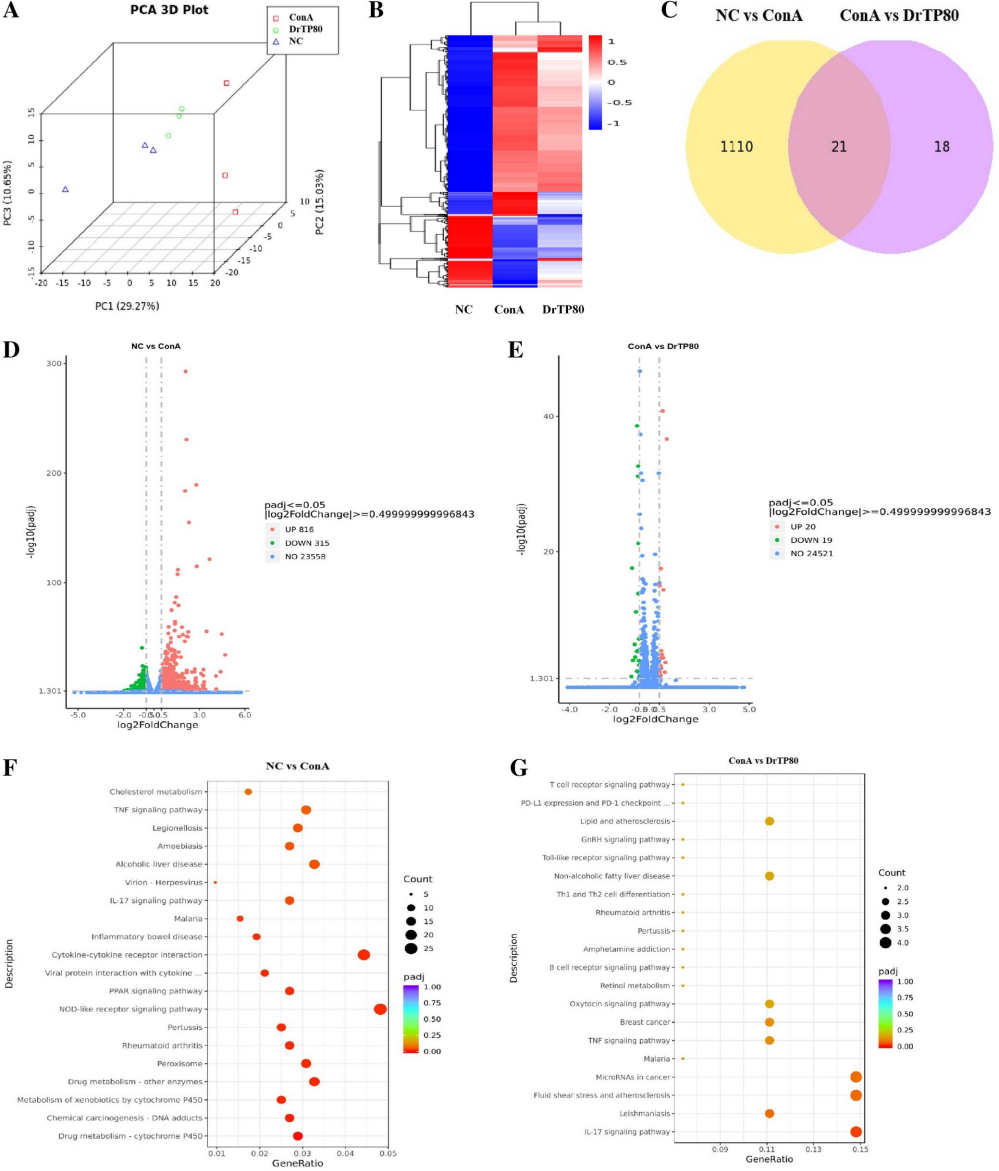
DrTP80 modulated the transcriptional expression. PCA score plots (A), heatmap (B), and Venn diagram (C) of samples from the NC, ConA, and DrTP80 groups. (D) Volcanic plots of samples from the NC and ConA groups. (E) Volcanic plots of samples from the ConA and DrTP80 groups. (F) KEGG pathway enrichment analysis of the NC and ConA groups. (G) KEGG pathway enrichment analysis of the ConA and DrTP80 groups. ConA, concanavalin A; KEGG, Kyoto Encyclopedia of Genes and Genomes; DrTP80, Polysaccharide 80 of *Dracocephalum tanguticum* Maxim; PCA, principal components analysis.

Principal component analysis (PCA) showed a distinct separation of gene expression patterns among the NC group, ConA group, and DrTP80 group (Figure 5A). Compared with the NC group, the ConA group had 11,131 differential genes (816 upregulated and 315 downregulated); compared with the ConA group, there were 39 differential genes (20 upregulated and 19 downregulated) in the DrTP80 group (Figure 5B–E). The functions of the differential genes were determined using KEGG pathway analysis (Figure 5F,G). Two key pathways were identified: TNF signaling pathway and IL‐17 signaling pathway.

**FIGURE 6:**
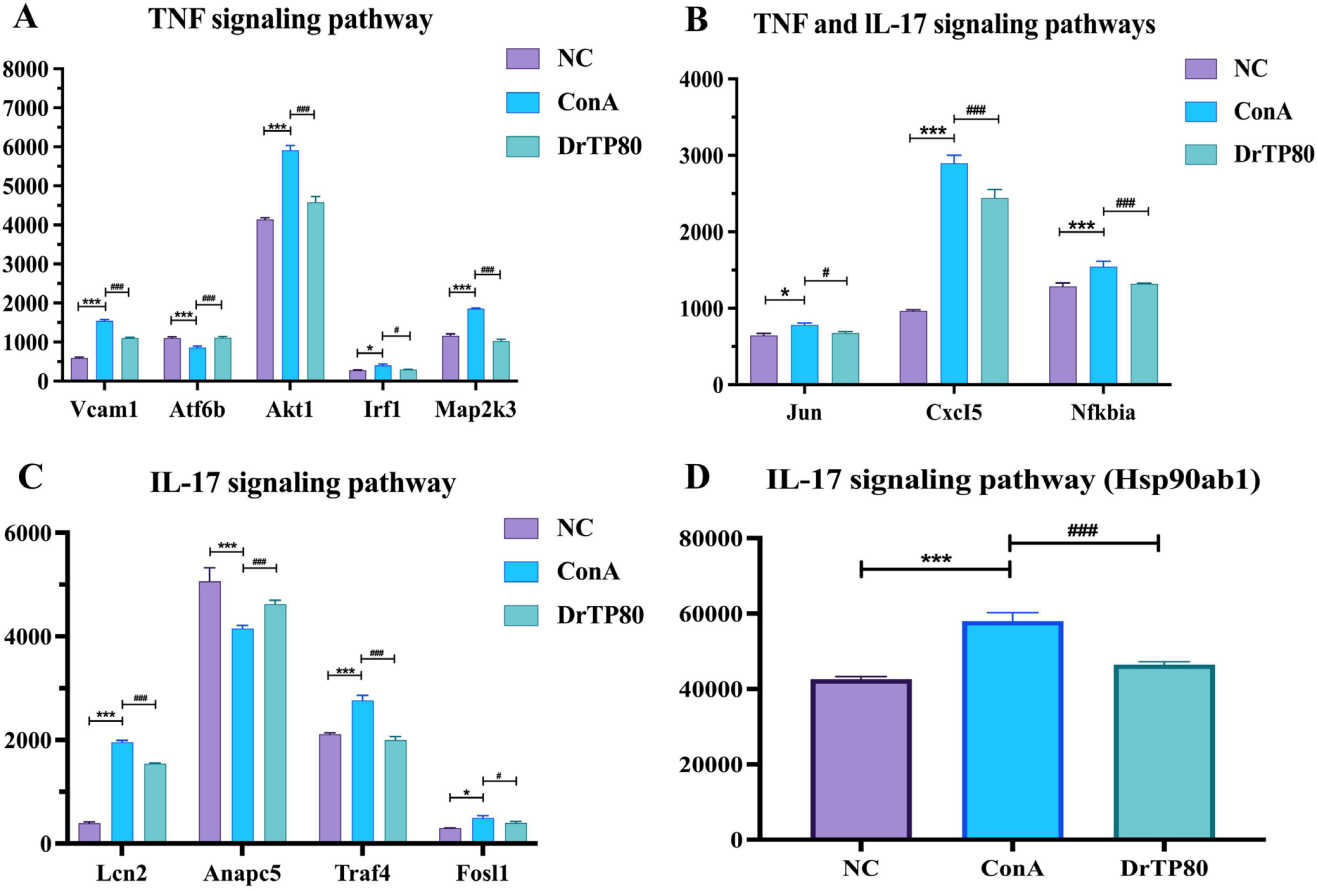
Differentially expressed genes in TNF and IL‐17 signaling pathways. All data are shown as mean ± SD (*n* = 3). *p < 0.05, ***p < 0.001 versus the NC group, ^#^ *p* < 0.05, and ^###^ *p* < 0.001 versus the ConA group. TNF, tumor necrosis factor; IL‐17, interleukin‐17.

Based on these two signaling pathways, 13 differentially expressed genes in NC, ConA and DrTP80 groups were screened, among which 5 genes (Vcam1, Atf6b, Akt1, Irf1, Map2k3) belonged to the TNF signaling pathway (Figure 6A), 5 genes (Lcn2, Hsp90ab1, Anapc5, Traf4, Fosl1) belonged to the IL‐17 signaling pathway (Figure 6C,D), and 3 genes (Jun, Cxcl5, Nfκbia) were shared by both pathways (Figure 6B).

#### 3.2.3 Results of Western Blot

**FIGURE 7:**
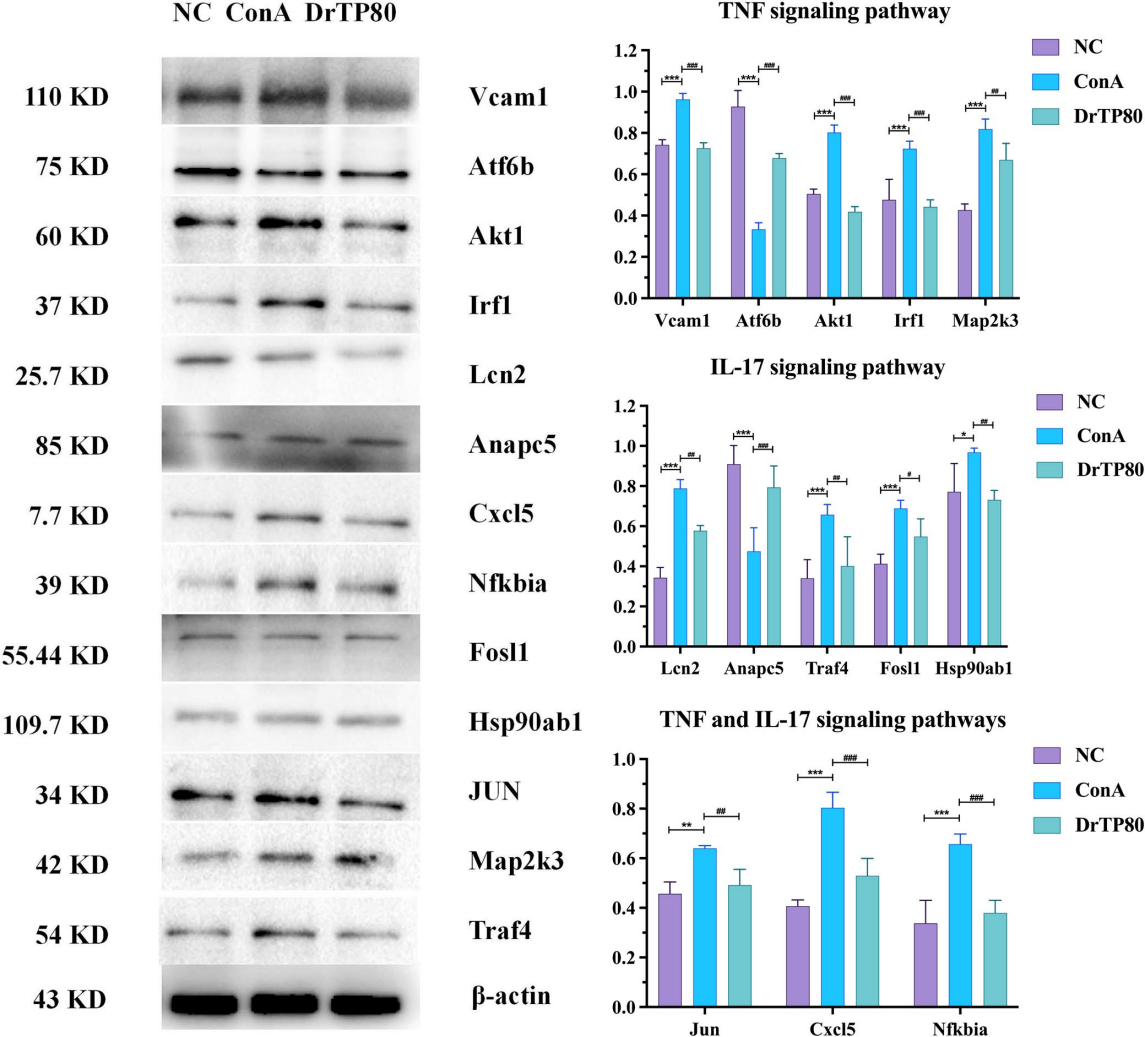
Quantification of Vcam1, Atf6b, Akt1, Irf1, Map2k3, Lcn2, Hsp90ab1, Anapc5, Traf4, Fosl1, Jun, Cxcl5, and Nfκbia expression. All data are shown as the mean ± SD (*n* = 3). *p < 0.05, **p < 0.01, and ***p < 0.001 versus the NC group; ^#^ *p* < 0.05, ^##^ *p* < 0.01, and ^###^ *p* < 0.001 versus the ConA group. Atf6b, activating transcription factor 6 beta; Cxcl5, chemokine (C‐X‐C motif) ligand 5; Fosl1, FOS‐like antigen 1; Hsp90ab1, heat shock protein ab1; Irf1, interferon regulatory factor 1; Lcn2, lipocalin 2; Map2k3, recombinant mitogen activated protein kinase kinase 3; Nfκbia, nuclear factor‐kappa inhibitor alpha; Traf4, tumor necrosis factor receptor‐associated factor 4; Vcam1, vascular cell adhesion molecule‐1.

Based on the transcriptome analysis results (Figure 7), we focused on TNF and IL‐17 signaling pathways, verified the levels of Vcam1, Atf6b, Akt1, Irf1, Map2k3, Lcn2, Hsp90ab1, Anapc5, Traf4, Fosl1, Jun, Cxcl5, and Nfκbia protein expression by Western blotting. The results were consistent with the transcriptome determination.

## 4 Discussion

Plant polysaccharides have strong potentials in protection against liver injuries (Dedhia et al. 2022; Yang et al. 2020; Cui et al. 2025). In the present study, we investigated the protective effect and mechanism of polysaccharides from *Dracocephalum tanguticum* Maxim in ConA induced mice and cells, aiming to provide a potential treatment for AIH.

Water‐soluble polysaccharides (DrTP40, DrTP60, and DrTP80) were obtained from *Dracocephalum tanguticum* Maxim using the water extraction‐ethanol precipitation method. There were abnormal aminotransferases and distinctive histological features in AIH (Muratori et al. 2023; Komori 2021). The serum aminotransferases and liver tissue morphology were studied (Hao et al. 2022; Covelli et al. 2021). The AST and ALT levels in the ConA group were significantly elevated, and the structure of hepatic lobules was disordered, accompanied by a large number of inflammatory infiltrates and cellular swelling. These biochemical indicators and pathological indicators revealed the successful induction of the AIH mouse model. DrTPs improved ConA‐induced liver injury to varying degrees, with DrTP80 showing the best effect. After treatment with DrTP80, the aminotransferases tended to normal, and the liver tissue morphology was significantly improved. Silibinin is a flavonoid compound extracted from plants, which has a history of more than 2000 years in the treatment of hepatobiliary diseases. In this study, it was chosen as the positive drug. Compared with it, DrTP80 can not only improve liver function, but also better relieve inflammation in liver tissue.

In this study, an immune liver injury cell model induced by ConA was established, and the protective effect of DrTP80 on it was investigated. 15 μg/mL of ConA could successfully induce the cell model, at this time, the cell activity was (81.3 ± 5.3)%. When the concentration of DrTP80 reached 250 μg/mL, it had a dose‐dependent protective effect on ConA‐induced hepatocyte injury.

The pathogenesis of AIH is not entirely unraveled, involving genetic variants, environmental factors, and epigenetic modifications (Sirbe et al. 2021; Webb et al. 2018). We determined the gene expression by RNA sequencing. Results showed that there were significant differences among the three groups, and the functions of differential genes were focused on TNF signaling pathway and IL‐17 signaling pathway. Based on these two signaling pathways, among which 5 genes (Vcam1, Atf6b, Akt1, Irf1, Map2k3) belonged to TNF signaling pathway (Figure 6A), 5 genes (Lcn2, Hsp90ab1, Anapc5, Traf4, Fosl1) belonged to IL‐17 signaling pathway (Figure 6C,D), and 3 genes (Jun, Cxcl5, Nfκbia) shared by both pathways. The ConA group showed higher expression of Vcam1, Akt1, Irf1, Map2k3, Lcn2, Hsp90ab1, Traf4, Fosl1 and lower expression of Atf6b, Anapc5. The expression levels were restored following treatment with DrTP80. According to previous studies, Vcam1, AKT1 and Lcn2 expression markedly increased in AIH mice (Li et al. 2023; Wang et al. 2022; Wang et al. 2023). Research showed that Irf1 was related to the evolution of various liver diseases (Chen et al. 2024), Knockdown of Map2k3 Negatively Regulates Hepatitis A Virus Replication (Kanda et al. 2021). Traf4, Fosl1 and Hsp90ab1 were prognostic biomarkers in hepatocellular carcinoma (Liu et al. 2017; Li et al. 2019; Vallejo et al. 2021; Xiao et al. 2021). Chemokines play a crucial role in the immunopathogenesis of liver cirrhosis and hepatocellular carcinoma, and may be diagnosis and prognosis biomarkers. CXCL5 may exacerbate nonalcoholic steatohepatitis in mice by regulating hepatocellular carcinoma cell migration (Laschtowitz et al. 2023; Qi et al. 2023). AnapC5 inhibited IL‐17‐mediated signal transduction, while its relationship with AIH still needs further study (Ho et al. 2013).

AIH is an autoimmune‐mediated liver disease characterized by chronic inflammation, and its pathogenesis involves multiple signaling pathways (Liu et al. 2022). Many inflammatory signals (such as TNF‐α and other pro‐inflammatory cytokines) can activate IKKs and translocate the ‐κB dimer from the cytoplasm to the nucleus through various signaling transduction, including MAPK, Akt, AP‐1 signaling pathways and so on, which ultimately leads to the synergistic expression of a variety of inflammatory and innate immune genes, forming an amplified cycle. There will also be crosstalk between signaling pathways, such as AP‐1 being regulated by the MAPK signaling pathway, and AKT1 also affects NF‐κB‐dependent gene transcription. Map2k3, Akt1, Fosl1, Jun, and Nfκbia, involved in this study, are key genes in these signaling pathways. CXC chemokines are particularly important for leukocyte infiltration in inflammatory diseases; Vcam1 and CXCL5 are produced after inflammatory cytokines IL‐1 and TNF‐α stimulate cells, while LCN2 and Irf1 participate in immunomodulation. TRAF4 is an adaptor protein that plays an important role in intracellular signal transduction, especially in the TNF receptor (TNFR) family, Toll‐like receptor (TLR) family, and IL‐1 receptor family. The above genes are closely related to the inflammatory response. Moreover, PNPLA5 participates in regulating the synthesis and decomposition of fatty acids and triglycerides and also affects the generation of signaling molecules by regulating phospholipase, thereby regulating the growth, differentiation, and apoptosis of cells. HSP90AB1 participates in multiple signaling pathways, influencing cellular survival or death by regulating the stability of apoptosis‐related proteins. ATF6B plays an important role in the cellular endoplasmic reticulum (ER) stress response. These results suggest that DrTP80 ameliorates AIH mainly via regulating the inflammatory response, while also regulating lipid metabolism and apoptosis.

## 5 Conclusion

Our study indicated that DrTP80 ameliorated autoimmune hepatitis both in vivo and in vitro via regulating TNF and IL‐17 signaling pathways. These results suggest that DrTP80 is a promising therapeutic candidate for AIH. This study provides a reference for the development and utilization of medicinal and edible plants and the treatment of AIH. Our further work will be focused on pur and structural characterization of DrTP80 which was screened out. Furthermore, the results were obtained from animal and cell experiments, and still needed to be verified in clinical trials.

## Author Contributions

**Min Guo:** experiments design and writing – original draft. **Saixue Wei:** performed the experiments. **Biaobiao Cheng:** performed the experiments. **Xiaodong Li:** experiments design, validation, writing – review and funding acquisition.

## Funding

This study was supported by the National Natural Science Foundation of China (No. 82160818) and the Science and Technology Plan Project of Lanzhou (No. 2025‐2‐146).

## Data Availability

The data that support the findings of this study are available on request from the corresponding author. The data are not publicly available due to privacy or ethical restrictions.
